# How useful are complete blood count and reticulocyte reports to clinicians in Addis Ababa hospitals, Ethiopia?

**DOI:** 10.1186/2052-1839-13-11

**Published:** 2013-12-11

**Authors:** Misganaw Birhaneselassie, Asaye Birhanu, Amha Gebremedhin, Aster Tsegaye

**Affiliations:** 1 Department of Medical Laboratory Sciences, Hawassa University College of Medicine and Health Science, Hawassa, Ethiopia; 2 School of Medical Laboratory Science, Addis Ababa University College of Health Science, Addis Ababa, Ethiopia; 3 Department of Internal Medicine, Addis Ababa University Medical Faculty, Addis Ababa, Ethiopia

**Keywords:** Complete blood count, Reticulocyte count, Differential leukocyte count, Clinicians, Laboratory report form, Clinical laboratory methods course

## Abstract

**Background:**

Complete blood count (CBC) and reticulocyte (Retics) are routine hematology tests useful for the differential diagnosis of anemia and other medical conditions. However, it has been presumed that they are not used as regular as they should be in medical practice in Addis Ababa hospitals.

**Methods:**

A hospital-based cross-sectional questionnaire survey was conducted during November-December 2010, in which 408 clinicians participated and their response on the use of CBC and Retics was assessed. The always/frequently (A/F) response was considered to reflect routine use of the CBC/Retics parameters by the clinicians. The Chi square test was used to study statistical associations among different variables.

**Result:**

Only four of 13 parameters in CBC were frequently or always used by more than 85% of the clinicians. Health Officers were observed to use 12 of the 13 CBC parameters less than the other professional group; interns and residents demonstrated highest use of CBC results. More than a third of clinicians’ preferred white blood cell (WBC) differential report in percentages than the more useful absolute number report. Reticulocyte parameters were not being used by majority of clinicians in patient management. Clinicians rated ‘average’ regarding the adequacy of clinical laboratory methods course they took during medical education. As service users, clinicians indicated mm^3^ as unit of preference in cell count on the laboratory report form.

**Conclusion:**

Overall, most clinicians do not use much of the data provided on routine CBC report. Additional research is needed to understand the issue further. Responsible bodies should promote the appropriate use of CBC/Retics reports by clinicians.

## Background

Clinical information obtained from laboratory tests play a key role in the diagnosis and management of patients [1]. A number of studies have shown that although physicians commonly request laboratory tests, they tend to use them for the wrong purposes, and ignore or misinterpret the results; such improper utilization and interpretation have obvious implications for the quality of patient care, and the economy as a whole [2,3].

The complete blood count (CBC) and reticulocyte (Retics) parameters are hematologic tests essential in the diagnostic workup of a broad variety of clinical conditions, mainly in the differential diagnosis of anemia and related disorders [4] which are commonly seen in Addis Ababa hospitals [5]. A study on women of reproductive age in Ethiopia reported that the leading causes of anemia are iron and vitamin B12 deficiency and chronic infections [5].

Clinicians do not always use objective data effectively, and the practice can potentially lead to increases in care costs as well as mortality and morbidity [6]. However, it is possible to correct flawed decision making due to inappropriate data utilization by a variety of approaches such as improving the laboratory form design, clinicians’ education in laboratory based course, and implementing feedbacks from clinicians [3,7,8].

In Ethiopia no documented data exists regarding the use and interpretation of hematological parameters in clinical practice. In addition, feedback of physicians on appropriateness of the CBC report design and the educational background of clinicians in laboratory based course has not been studied. This study aims at ascertaining the extent of use of CBC/Retics results, and feedback of clinicians on CBC in Addis Ababa hospitals.

## Methods

A cross-sectional, questionnaire-based survey was conducted in Addis Ababa hospitals in November and December 2010. Clinicians involved in the diagnosis and treatment of patients in Addis Ababa hospitals, including interns, residents, health officers (*HO), general practitioners (GP), and specialists participated in the survey*.* Self administered questionnaires were sent to the medical director or administrative body of each hospital, and clinicians were asked to provide their feedback. [*HO: Health Officers in Ethiopia are medical graduates who undertake promotive, preventive, curative and rehabilitative services compatible to the needs of the population; are public health oriented and have less years of training than medical doctors].

The questionnaire used in the survey asked physicians to rate the frequency of use of each component of the CBC/differential and reticulocyte reports for patient management. Items included in a CBC test report were white blood cell count (WBC), WBC differential, hemoglobin (HGB), hematocrit (HCT), red blood cell (RBC) count, mean cell volume (MCV), mean cell hemoglobin (MCH), mean cell hemoglobin concentration (MCHC), red cell distribution (RDW), platelet (PLT) count, mean platelet volume (MPV), platelet distribution width (PDW) and morphologic comments on RBC and WBC. *[Various automated hematology analyzers of different manufacturers are used in Addis hospitals: Cell Dyn 1800 and Sysmex are used commonly for CBC; Abbot Cell Dyn 3200, Beckman Coulter LH 500 and* Sysmex***-***2000 x*i are used for CBC and Retics].* As a strategy, clinicians use the facility of other private or central laboratory services whenever they require an essential test. Reticulocyte reports included were the reticulocyte percentage, reticulocyte absolute count, reticulocyte production index (RPI) and the corrected reticulocyte count.

Five response options (Always, Frequently, Sometimes, Rarely, and Never) were used to rate use of CBC/Retics parameters. The always/frequently response was considered to be adequate or to reflect routine use of the CBC/Retics parameter by the clinicians. Physicians were also asked to indicate their preferences of differential reports either in percent, absolute count or both, and provide their opinion about the amount of data contained in the CBC reports (too little, too much, or just right), and preferred unit of measurement in cell count. Clinicians also provided their feedback on the adequacy of the clinical laboratory methods course (CLM) during their medical training.

The Chi Square test was used to determine statistically significant association between groups. SPSS statistical package version 17 was used for data analysis. The study proposal was defended in the school of Medical Laboratory Sciences and approved by the Institutional Review Board (IRB) of Addis Ababa University, School of Medicine. The Addis Ababa Health Bureau, the administration and the Medical Director Office of the hospitals were requested for permission and cooperation to conduct the survey. Individual consent was obtained from all participants.

## Result

### Study participants

The survey assessed the frequency of use of CBC and reticulocyte reports by a total of 408 clinicians from 34 public and private hospitals in Addis Ababa. The response rate was 100%. Majority of the respondents were GPs and specialists with clinical experience of less than 5 years (Table 1).

**Table 1:** Distribution of study participants in Addis Ababa hospitals, from Nov 2010-Dec 2010

**Category of participants**	**Frequency**	**Percentage**
**Institution (n = 48)**		
Private	126	31%
Government	282	69%
**Profession (n = 408)**		
*HO	97	24%
GP	128	31%
Specialist	120	29%
Intern	13	3%
Resident	50	12%
**Years of experience (n = 408)**		
<5 yrs	234	58%
6-10 yrs	75	18%
> 10 yrs	99	24%
**Specialty (n = 120)**		
Int Medicine	33	28%
Gyn-Obs	28	24%
Surgery	15	13%
Pediatrics	24	20%
Others*	20	15%

*HO: Health Officers.

*Others: Specialists working in other areas of specialty other than listed.

### Complete blood count

Only 4 of the 13 parameters in the CBC (hemoglobin, haematocrit, WBC count, and WBC differential) were frequently or always used by more than 85% of the physicians (Table 2). Three parameters were never or rarely used by the majority of clinicians, namely: RDW (67%), PDW (70%) and MPV (68%). Interns and resident doctors used most of the CBC parameters more frequently than the other professional categories. HO’s showed lesser ratings to more component of the CBC (12/13) than the other professional group.

**Table 2:** Rate of use of CBC parameters by profession in Addis Ababa hospitals Nov 2010-Dec 2010

	**RBC count**		**HGB**		**HCT**		**MCV**		**MCH**		**MCHC**		**RDW**		**Cell morphology**		**PLT count**		**MPV**		**PDW**		**WBC count**		**WBC**	**Diff**
**Profession**	**F/A**	**R/N**	**F/A**	**R/N**	**F/A**	**R/N**	**F/A**	**R/N**	**F/A**	**R/N**	**F/A**	**R/N**	**F/A**	**R/N**	**F/A**	**R/N**	**F/A**	**R/N**	**F/A**	**R/N**	**F/A**	**R/N**	**F/A**	**R/N**	**F/A**	**R/N**
GP (n = 128)	59	19	91	<1	90	2	28	24	24	29	22	32	11	66	15	23	63	8	10	69	12	67	98	<1	96	0
HO (N = 97)	41	14	73	2	77	3	16	2	13	45	12	47	2	74	19	31	39	24	5	68	3	77	87	0	81	5
Intern (n = 15)	77	15	100	0	92	0	39	0	31	0	31	8	15	62	23	8	92	8	15	62	15	62	100	0	85	15
Resident (n = 50)	64	10	94	0	98	0	50	16	44	18	32	32	8	56	30	28	72	8	10	62	6	58	94	2	96	2
Specialist (n = 120)	43	29	89	3	93	<1	38	29	35	30	33	31	15	68	19	26	58	9	12	73	11	73	93	0	88	0
All (n = 408)	**52**	**20**	**87**	**2**	**89**	**1**	**31**	**28**	**27**	**31**	**24**	**35**	**10**	**67**	**19**	**26**	**58**	**12**	**10**	**68**	**9**	**70**	**93**	**<1**	**90**	**2**

F/A, (Frequently or always use); RN (rarely or never use).

Response rates are given in percentages. The “sometimes” response rate (not shown) can be calculated by using the following equation: 100%-[F/A + R/N].

**WBC**: white blood cell, **RBC**: red blood cell, **HGB**: hemoglobin, **HCT**: hematocrit, **MCV**: mean cell volume, **MCH**: mean cell hemoglobin, **MCHC**: mean cell hemoglobin concentration, **RDW**: Red cell distribution width, **PLT**: plate let, **MPV**: mean platelet volume, **PDW**: Platelet distribution width.

WBC differential was reported to be used always or frequently by 90% of the clinicians. Slightly less than half of the clinicians (46%) preferred WBC differential to be reported in absolute numbers and percentages, 39% in percentages only and 15% in absolute numbers only.

Some of CBC parameters have significant association among the professional group: RBC (p < 0.001), MCV (p < 0.001), MCH (p < 0.001), PLT (p <0.001), MCHC (P < 0.01) and RDW (p < 0.05). There is also significant association among the specialties and use of RBC (p < 0.01), MCHC (P < 0.05), and MCV, MCH (p < 0.001). Internists highly rated use of CBC parameters than the other specialist counterpart (Table 3).

**Table 3:** Rate of use of CBC parameters by specialty in Addis Ababa hospitals, Nov 2010-Dec 2010 (N = 120)

	**RBC count**		**HGB**		**HCT**		**MCV**		**MCH**		**MCHC**		**RDW**		**Cell morphology**		**PLT count**		**MPV**		**PDW**		**WBC count**		**WBC**	**Diff**
**Specialty**	**F/A**	**R/N**	**F/A**	**R/N**	**F/A**	**R/N**	**F/A**	**R/N**	**F/A**	**R/N**	**F/A**	**R/N**	**F/A**	**R/N**	**F/A**	**R/N**	**F/A**	**R/N**	**F/A**	**R/N**	**F/A**	**R/N**	**F/A**	**R/N**	**F/A**	**R/N**
Gyn-Obs (n = 28)	25	43	93	7	96	0	15	56	14	54	11	56	7	93	19	35	36	18	11	86	7	93	82	0	71	0
Int Medicine (n = 34)	73	12	97	0	100	0	73	9	67	9	58	12	24	49	31	13	88	3	21	58	22	50	100	0	100	0
Surgery (n = 15)	40	27	100	0	93	0	27	33	20	40	27	40	13	67	0	43	60	7	13	60	13	67	100	0	100	0
Pediatrics (n = 24)	33	33	70	4	88	0	42	12	46	17	46	17	21	50	8	8	42	12	4	75	4	75	96	0	92	0
Others (n = 20)	35	35	85	5	85	5	10	40	10	40	10	40	5	85	25	45	60	5	5	85	5	85	85	0	75	0
All (n = 120)	**43**	**29**	**89**	**3**	**93**	**<1**	**38**	**29**	**35**	**30**	**33**	**31**	**15**	**67**	**19**	**26**	**58**	**9**	**12**	**72**	**11**	**73**	**93**	**0**	**88**	**0**

F/A, (Frequently or always use); RN (rarely or never use).

Response rates are given in percentages. The “sometimes” response rate (not shown) can be calculated by using the following equation: 100%-[F/A + R/N].

**WBC**: white blood cell, **RBC**: red blood cell, **HGB**: hemoglobin, **HCT**: hematocrit, **MCV**: mean cell volume, **MCH**: mean cell hemoglobin, **MCHC**: mean cell hemoglobin concentration, **RDW**: Red cell distribution width, **PLT**: plate let, **MPV**: mean platelet volume, **PDW**: Platelet distribution width.

### Reticulocyte count

Most clinicians rated use of reticulocyte parameters, rarely or never. Out of the clinicians who use the parameter, 52% preferred to receive the reports as percentages while 41% preferred absolute numbers (Table 4).

**Table 4:** Rate of use of reticulocyte parameters by clinicians in Addis Ababa hospitals, Nov 2010-Dec 2010 (n = 408)

**Profession**	**Retics %**		**Retics Abs**		**RPI**		**Corrected Retics**	
	**A/F**	**R/N**	**A/F**	**R/N**	**A/F**	**R/N**	**A/F**	**R/N**
GP (n = 128)	8	41	7	59	4	75	2	74
HO (n = 97)	9	66	5	68	4	84	5	79
Intern (n = 13)	23	23	15	23	0	31	8	39
Resident (n = 50)	10	40	8	44	6	64	6	60
Specialist (n = 120)	10	47	5	64	3	77	2	75
**Total (n = 408)**	**10**	**48**	7	**59**	**4**	**75**	**4**	**72**

F/A, (frequently or always use); R/N, (rarely or never use). Response rates are given in percentages. The “sometimes” response rate (not shown) can be calculated by using the following equation: 100%-[F/A + R/N].

### Feedback of clinicians and adequacy of CLM course

Majority of the clinicians preferred mm^3^ as units of measurement of cell count. Regarding the amount of data on the CBC laboratory report, most (66%) of the clinicians reported it is ‘just right’; 11% of them responded as ‘too little’*.* Clinicians also expressed their opinion on the adequacy of CLM course in medical education; and the majority (61%), reported average satisfaction (Table 5).

**Table 5:** Feedback of clinicians in Addis Ababa hospitals on CBC and adequacy of CLM course from Nov 2010-Dec 2010

**Parameter**	**Frequency**	**Percent (%)**
**CBC content**		
(n = 399)		
Just right	262	66%
Too much	91	23%
Too little	46	11%
**Unit for cell count**		
(n = 396)		
Per mm^3^	264	66%
Per μL	110	28%
Per L	22	6%
**Adequacy of CLM course**		
(n = 391)		
Excellent and very good	127	32%
Good and fair	226	58%
Poor and bad	38	10%

## Discussion

This study showed that the use of CBC and reticulocyte results for clinical decision making in patient management in Addis Ababa hospitals was inadequate. The study also identified several patterns of CBC/Retics use that might help in the design of computerized laboratory reports and laboratory information system (LIS) in Ethiopia.

### Complete blood count

The red cell indices: The study indicated that HCT and HGB were the most frequently used red cell measurements by the majority of clinicians. Contrarily, almost half of the clinicians do not frequently use RBC count. Other studies have also reported that RBC count is not used commonly in decision making [9].

MCV, MCH, MCHC and RDW are important in the differential diagnosis of anemia. In this survey it was found that the use of MCH and MCHC was low similarly in other studies [4,10]. MCV is regarded as the single most useful and critical erythrocyte index in the evaluation of anemia [9,10]. Nevertheless, this survey revealed that only a third of the clinicians used this key parameter frequently in the classification of anemia. It is a daily practice to treat anemia in Addis Ababa hospitals [5], and despite the fact that the first step in approaching anemia is to classify the process as microcytic, normocytic or macrocytic using this valuable diagnostic tool, clinicians in Addis Ababa have differently very low use of the MCV [4,10].

RDW was found to be used even much less frequently compared with other red cell indices. In another study, it has been shown that the majority of the clinicians [10] reported to use RDW always or frequently; however, it has been also reported that RDW is a largely overlooked parameter [11].

Among the professionals, interns and residents demonstrated a highest rate of use which is assumed more of superfluous [7,12]. MCV was rated to be used by 38% of Specialists > 28% of GPs and > 16% of HOs. RDW was reported to be used highest by specialists (15%) > GPs (11%) > HOs (2%). This difference can be because specialists are geared to look for differential diagnoses; which orient them toward laboratory workups [12] than GPs and HOs [13,14]. Moreover, MCV was used more frequently in internal medicine departments as internists tend to treat more cases of anemia and other internal disorders compared to specialists in other categories.

#### Cell morphology comments

Obtaining a peripheral blood smear (PBS) during the initial evaluation of anemia has been recommended by other researchers as it gives additional clues from morphologic features of RBC and WBCs which substantially enhances the initial process of differential diagnosis and provides guidance for further testing [4]. This survey found only 19% of the clinicians use RBC morphology in most of the cases, while the Sandhaus study found 52% of the clinicians use RBC morphology always and frequently for anemia diagnosis and differentiation [10].

#### Platelet parameters

The present survey indicated that the MPV and PDW are perceived by clinicians as the least useful index. Only less than 10% of the clinicians reported that they use the MPV or PDW frequently or always in medical practice. In spite of the clinical benefits of platelet parameters, the contribution of the MPV and PDW to clinical practice in Addis Ababa hospitals was low.

#### WBC count, WBC differential and unit of reporting

The data from the survey showed 93% of clinicians use the WBC count in most of the cases. Likewise, 90% of the clinicians use the WBC differential mostly in medical practice. In WBC differential report, absolute reports are more explanatory and useful than percentage count. The present study showed that more than a third of the clinicians prefer percentages of WBC differential than the more useful absolute numbers. This indicates that a significant number of the clinicians interpret differential reports incorrectly, and may not be obtaining the valuable information required, which could mislead diagnosis.

### Reticulocyte count

The use of the reticulocyte parameter in Addis Ababa hospitals is quite low. Lack of hematology analyzers that perform both CBC and Retics in most hospitals can largely limit Retics use; yet as a strategy clinicians could use extra laboratory services when additional investigations are required. The use of reticulocyte count as percentage and absolute unit assessed from the frequency of use of reticulocyte parameter indicated that slightly more than half of the clinicians claimed to use reticulocyte count in percentage units. Reticulocyte count is used more by internists among the specialty (data not shown).

### Feedback of clinicians on complete blood count

Units of measurement: Cell counts such as RBC, PLT and WBC can be reported per mm^3^, per μL, or per L. This study showed that most of the clinicians prefer cell count to be reported in mm^3^ unit. This implies that emphasis should be given in the reporting systems during medical training thus clinicians are well acquainted with the use of the international standard (SI) measurement units [15,16].

#### Amount of data

Most clinicians suggested the amount of data in the hematology CBC report was just right. Only about one in ten respondents mentioned that the amount of data was inadequate and most of these were internists. This phenomenon was also reflected in internists’ frequent use of reticulocyte reports, which further suggests that there is a need for additional laboratory tests for use by a segment of medical specialties (internists). Although more dozens of hematology parameters useful for patient management are not part of the CBC reports of Addis Ababa hospitals, majority of the clinicians are satisfied with the limited CBC parameters available from the common hematology analyzers. In fact use of more advanced parameters parallels expansion of the health standard and service within the country.

#### Clinical laboratory methods course

The results of this study indicated that most clinicians were not satisfied with the quality of their CLM course, although more than a quarter rated it as ‘excellent’ or ‘very good’. This shows that there is room for improvement in the course, both in terms of quality and quantity of materials covered.

## Conclusion

CBC/reticulocyte parameters are important hematological tests used routinely in clinical decision making mainly in relation to anemia and blood disorders. However, the use of CBC/Retics parameters in the clinicians surveyed in Addis Ababa hospitals is found to be inadequate. The study suggested that laboratory based medical course (CLM), and content of a CBC report form contributed to the low use rate of these parameters. Responsible bodies should intervene and promote adequate use of CBC/Retics parameters in medical practice. Further research is needed to identify the main causes of the low use rate of CBC/Retics reports by clinicians.

## Competing interests

The authors declare that they have no competing interests.

## Authors’ contributions

MB was the principal investigator for the study; MB, AB, AG and AT contributed to the design of the study. MB carried out the data collection; AB and AT supervised data collection; MB, AG and AT interpreted the result; all authors approved the final manuscript.

## Pre-publication history

The pre-publication history for this paper can be accessed here:

http://www.biomedcentral.com/2052-1839/13/11/prepub
